# Divergent profiles of rhizosphere soil carbon and nitrogen cycling in *Pinus massoniana* provenances with different types of carbon storage

**DOI:** 10.3389/fmicb.2025.1537173

**Published:** 2025-03-17

**Authors:** Zichen Huang, Jiannan Wang, Xin He, Mengyang Zhang, Xingyue Ren, Wenya Yu, Sheng Yao, Kongshu Ji

**Affiliations:** ^1^State Key Laboratory of Tree Genetics and Breeding, Co-Innovation Center for Sustainable Forestry in Southern China, Nanjing Forestry University, Nanjing, China; ^2^Key Laboratory of Forestry Genetics and Biotechnology of Ministry of Education, Nanjing Forestry University, Nanjing, China; ^3^Co-Innovation Center for Sustainable Forestry in Southern China, Nanjing Forestry University, Nanjing, China; ^4^College of Biology and the Environment, Nanjing Forestry University, Nanjing, China

**Keywords:** *Pinus massoniana*, provenance, soil metagenomics, carbon and nitrogen cycling, functional genes

## Abstract

**Introduction:**

In subtropical China, *P. massoniana* is a timber tree species which have a great potential for carbon sequestration. However, few studies have investigated how varying levels of carbon storage in *P. massoniana* provenances affect the soil microbial functional potential related to nutrient cycling within the rhizosphere.

**Methods:**

In this investigation, metagenomic sequencing was employed to explore the differences in carbon and nitrogen cycling capabilities among rhizosphere microbial communities within *P. massoniana* provenances, categorized by high, medium, and low levels of carbon storage.

**Results:**

Our findings revealed a significant increase in the relative abundance of *Acidobacteriota* and *Ascomycota* by 23 and 61%, respectively, whereas *Basidiomycota* significantly decreased by 8% in the rhizosphere of *P. massoniana* provenances with high carbon storage compared with those with low carbon storage. The variability in carbon storage among *P. massoniana* provenances was linked to marked disparities in the presence of key genes essential for carbon and nitrogen cycling within their rhizosphere soils.

**Discussion:**

Notably, in *P. massoniana* provenances characterized by high carbon storage, the rhizosphere presented a significantly elevated presence of genes associated with carbon decomposition, carbon assimilation, methane generation, and denitrification, in stark contrast to provenances with medium and low carbon storage. Furthermore, *P. massoniana* provenances with high carbon storage rates presented increased transformation and availability of soil carbon and nitrogen, along with increased potential for ecological restoration. Moreover, the rhizosphere soil nitrification of *P. massoniana* provenances with low carbon storage surpassed that of other provenances, leading to increased available nitrogen content and elevated nitrate leaching risk. In the *P. massoniana* rhizosphere, critical soil factors, including soil organic carbon (SOC), total nitrogen (TN), pH, and nitrate nitrogen (NO_3_^−^-N) content, significantly shape the functionality of genes associated with carbon and nitrogen cycling. In conclusion, our study lays a scientific foundation for establishing *P. massoniana* plantations and identifying *P. massoniana* provenances with superior ecological value and potential.

## 1 Introduction

As principal terrestrial ecosystems, forests are essential for the sequestration of CO_2_ and the release of O_2_, thereby sequestering approximately two-thirds of the organic carbon present in land-based ecosystems (Song et al., 2012). The forest ecosystem contains approximately 85% of the terrestrial biomass and 40% of the global soil carbon storage (Zhou et al., 2000). The vegetation in forests can sequester 2.4 petagrams of carbon (Pg C) from the atmosphere annually through photosynthesis (Pan et al., 2011). The shift in global climate patterns has increasingly drawn attention to alterations in carbon storage within forest ecosystems. Simultaneously, an increasing volume of research is being conducted to explore the link between terrestrial biotic carbon sequestration and the influencing factors of soil ecology, with additional emphasis on the rhizosphere microenvironment (Heinrich et al., 2021; Jiao et al., 2017; Labriere et al., 2023).

*Pinus massoniana* Lamb., belonging to the genus *Pinus* within the Pinaceae family, stands out as a prominent coniferous tree species in southern China (Liu, 2016). This species has a wide distribution, robust environmental adaptability, rapid growth rate, and significant economic and ecological value. According to the findings of the ninth forest inventory, the natural forest volume of *P. massoniana* was measured at 586.999 million cubic meters, whereas the planted forest volume was 321.294 million cubic meters, ranking third among tree species, behind poplar and Chinese fir (Ji et al., 2022). The carbon storage capacity of *P. massoniana* is at the upper level in planted forests in southern China (Du et al., 2014). A carbon storage estimation of *P. massoniana* plantations located in Guangxi, China, was conducted. The results indicate an average carbon sequestration of 87.67 tons per hectare, significantly surpassing the national forest average in China (Xu et al., 2016). Given its substantial forest carbon storage capacity, *P. massoniana* is crucial for regional carbon cycling and adaptation to climatic alterations. Some studies have reported variations in the carbon sequestration capacity or biomass of *P. massoniana* across different families or provenances (Hu et al., 2022b). Earlier studies have focused predominantly on estimating the aboveground carbon storage and biomass of *P. massoniana*. In recent years, researchers have begun to link the aboveground carbon storage of *P. massoniana* with belowground parts, such as soil properties and rhizosphere microorganisms. Substantial variability was noted in the community structure and carbon metabolic profiles of the soil microbes within the rhizosphere across the different *P. massoniana* provenances, which differed in their carbon storage levels. These differences were found to be influenced by rhizosphere effects and were correlated to soil physicochemical properties (Huang et al., 2023).

Encompassing plant roots, the rhizosphere consists of a confined area that harbors various microbial populations and functions as an interaction microenvironment among the soil, roots, and microbes (Xiong et al., 2020). In the rhizosphere, the interplay between root activities and the microbial consortia critically influences soil physicochemical attributes, thereby affecting the dynamics of microbial communities (Zhao et al., 2023). Approximately 21% of the carbon captured via photosynthesis is exuded into the rhizosphere by plant roots as soluble sugars and various secondary compounds that are subsequently utilized by the microbial community within the rhizosphere (Chaparro et al., 2013; Huang et al., 2014). They found that the extent of the rhizosphere effect varies depending on the plant species. Research has demonstrated a close relationship between plant nutrient absorption, photosynthetic activity, and the condition of the rhizosphere (Schulze et al., 1994). Typically, plants with greater photosynthetic (carbon fixation) capacity tend to accumulate more assimilates in their roots and exhibit a concurrent increase in root exudate production, leading to enhanced rhizosphere effects. This process may further impact the cycling of carbon and nitrogen by rhizosphere microbes (Ma et al., 2023).

Carbon and nitrogen cycling within the soil is a fundamental biogeochemical mechanism that is critical for maintaining soil quality, enhancing agricultural productivity, and moderating atmospheric gas exchanges (Hu et al., 2022a). Microorganisms are pivotal in these processes and participate in multiple phases of carbon and nitrogen turnover within soil ecosystems. Their activities include the decomposition of organic material, the fixation of nitrogen, and nitrification and denitrification processes. Their activities are influenced by numerous factors, including the soil organic matter content, pH, and plant type, all of which can impact microbial growth and metabolic functions (Huang et al., 2014; Wang et al., 2022). The extraction and sequencing of microbial metagenomes offer valuable insights into the structural and functional frameworks of microbial assemblies in the rhizosphere, thus overcoming the limitations imposed by conventional microbial cultivation experiments (Lombard et al., 2011). Certain findings have indicated that the use of organic fertilizer significantly reduces the abundance of *COXS* (carbon monoxide oxidation) genes and *COOC* (reduced acetyl-CoA pathway) genes while increasing the presence of *ICD* (RTCA cycle) genes and genes related to soil nitrogen degradation, nitrification, and anammox within the dark soil area of China’s northeastern sector (Hu et al., 2022a). Investigations into the dynamic composition and functional potential of microbial ecosystems involved in nutrient cycling throughout different growth phases of fir plantations revealed an increase in the abundance of cellulose degradation pathways as stand age progressed (Wang et al., 2022). The fluctuations in the relative abundance of key microbial genes under varying environmental conditions and the regulation of these factors can enhance microbial activities and optimize soil carbon and nitrogen cycling efficiency.

Our prior research in this area, which utilized 16S/ITS amplicon fragment sequencing, revealed notable variances in the composition and predominant species within bacterial and fungal communities across the rhizosphere of *P. massoniana* provenances that varied according to their carbon storage capacities (Huang et al., 2023). This research provides crucial perspectives on the soil microbial diversity and community composition within *P. massoniana* plantations. Nevertheless, thorough insights into microbial metabolic functions and capabilities continue to be urgently needed. The objective of this research was to elucidate the genetic capabilities of microorganisms involved in carbon and nitrogen cycling within soil ecosystems. This was achieved by analyzing the structure of microbial communities and identifying functional genes related to the cycling of carbon and nitrogen within the rhizosphere of *P. massoniana* via a metagenomic approach. Our goals were (1) to discern the variances in the soil microbial community structure within the rhizosphere of *P. massoniana* provenances, distinguished by their diverse carbon storage, (2) to investigate the functional repertoire of soil microorganisms that are involved in the processing of carbon and nitrogen cycles in the rhizosphere of *P. massoniana* provenances characterized by varied levels of carbon storage, and (3) to elucidate the primary factors shaping the soil microbial carbon and nitrogen cycling within the rhizosphere of *P. massoniana* plantations. Overall, this study sought to elucidate the functions of soil microbial communities within carbon and nitrogen cycles as well as their responses to variations in *P. massoniana* provenance carbon storage. These findings could serve as a theoretical foundation for establishing *P. massoniana* plantations with significant carbon storage capacity.

## 2 Materials and methods

### 2.1 Experimental design

In July 2022, samples were collected at the State-owned Forest Seed Farm in Yu’an District, Lu’an city, Anhui Province, China. The coordinates of this area are 116°12′ E, 31°40′ N. The region is situated 80–100 m above sea level and has a subtropical monsoon climate. The average annual temperature is 15°C, accompanied by annual precipitation totaling 1239.8 mm, with a frost-free period of nearly 225 days. The identified soil is characterized as clayey, with a pH that marginally leans toward acidity. The *P. massoniana* plantation was established in the spring of 1,981 and is comprised of a total of 64 provenances were arranged according to a completely randomized block experimental layout. The experimental forest is currently 43 years old, and the survival rates of some provenances fail to meet statistical requirements because of growth competition and pest and disease impacts. Currently, 55 provenances continued to satisfy the statistical benchmarks.

We previously measured the tree diameter at crown height and breast height (DBH) of the *P. massoniana* provenance in the experimental stands. Biomass was estimated via the binary equation model specified in the Chinese forestry industry standard (LY/T2263-2014). The carbon storage values of each plant organ were calculated by multiplying the biomass with its respective carbon content coefficient. The total carbon storage per plant of the provenance was then summed. Three *P. massoniana* provenances, San Ming (SM), Qing Jiang (QJ), and He Feng (HF), were chosen to represent high, medium, and low levels of carbon storage, respectively. For each provenance, four *P. massoniana* trees were identified, each exhibiting a diameter at breast height (DBH) and crown height within 10% of the mean values. The litter layer under each plant was removed, and soil samples were taken at a depth of 30 cm, 1 m from the tree trunk. Rhizosphere soil was collected from first-order fine roots (the most physiologically active very fine roots) with a root diameter values of 0–0.5 mm. A portion of the soil was utilized for DNA extraction, while the rest was analyzed for soil physicochemical properties and enzyme activity.

We measured the soil total nitrogen (TN), soil organic carbon (SOC), soil pH, nitrate nitrogen (NO_3_^−^-N), and ammonium nitrogen (NH_4_^+^-N) contents. Methodological specifics for evaluating soil physical and chemical properties are detailed in Supplementary material Text S1. The results of soil properties have been described in our previous study (Huang et al., 2024) and summarized in Supplementary Table S1.

### 2.2 Determination of soil enzyme activity

The activities of the four enzymes were studied in freshly collected soil samples. These enzymes include two carbon cycle enzymes, polyphenol oxidase (PPO) and peroxidase (POD), which are involved primarily in refractory lignin degradation, and two nitrogen cycle enzymes, urease (UR) and nitrite reductase (NiR), which are involved in nitrogen degradation within the nitrogen cycle (Saiya-Cork et al., 2002). The POD Activity Assay Kit, PPO Activity Assay Kit, UE Activity Assay Kit, and NiR Activity Assay Kit used in our study were all procured from Crisp Biotechnology (shanghai) Co., Ltd. After processing and incubating the samples according to the kit instructions, the supernatant was collected, and the absorbance at 475 nm was measured to assess PPO and POD enzyme activities. Absorbance values at 578 nm and 540 nm were used to detect UR and NiR enzyme activities, respectively.

### 2.3 Metagenomic sequencing and data processing and annotation

DNA was extracted from the soils collected for microbial analysis via the QIAamp® Fast DNA Stool Mini Kit (Qiagen, Hilden, Germany). The assessment of DNA purity and structural integrity was conducted using agarose gel electrophoresis and a NanoDrop2000 spectrophotometer (Thermo Fisher Scientific, Waltham, MA, United States). Fragmentation of the DNA was achieved through the application of S220 focused ultrasonicators (Covaris, United States), followed by a step for purification utilizing Agencourt AMPure XP beads (Beckman Coulter Co., United States). For library construction, the TruSeq Nano DNA LT Sample Preparation Kit (Illumina, San Diego, CA, United States) was used, followed by sequencing via the Illumina NovaSeq 6000 platform. The project can be accessed in NCBI under BioProject ID PRJNA1192390. The raw FastQ data were processed with fastp (v 0.20.1) to remove low-quality bases and reads containing ambiguous bases (N bases). The double-end reads were aligned with the host genome using bbmap (v38.93-0) and this part of the sequence was removed. Valid reads were then subjected to metagenome assembly via MEGAHIT (v 1.2.9). We used Prodigal (v 2.6.3) to predict the ORFs in the assembled scaffold, which were then converted into amino acid sequences. For all samples, we constructed a nonredundant gene set from the predicted genes, employing MMSeqs2 (version 13.45111), and adjusted the clustering parameters to require 95% sequence identity and coverage of at least 90%. Within each cluster, the gene with the greatest length was designated the representative sequence. Salmon (v 1.8.0) subsequently compared the clean reads against a nonredundant gene set (95% identity) and estimated the abundance of genes within each sample. For statistical analysis, the relative abundance of genes was calculated using the transcripts per million (TPM) formula (Xie et al., 2022). The formula is as follows:


$$TPM=N_{g}/{L_{g}}^{*}\left(1/\sum j\left(N_{j}/L_{j}\right)\right)*10^{6}\text{.}$$

*N* is the number of reads aligned to the gene; *L* is the length of the gene; *g* is the index of the specific gene; and *j* is the index of the gene set.

Annotation of species was derived from the taxonomic NR library, with the relative abundance of each species being estimated from gene relative abundances. Gene sets representative amino acid sequences were analyzed against the KEGG database via DIAMOND (v 0.9.10.111) software.

### 2.4 Statistical analysis

Carbon and nitrogen cycling-related genes were identified on the basis of a literature review of key pathways found in all metagenomic samples. Gene-associated relative abundance was determined by comparing all KEGG KOs. Details of the selected functional genes are presented in Supplementary Table S1. Duncan’s test (*p* < 0.05) was used for the identification of statistically significant differences. To explore the associations between environmental variables and microbial functional genes, both Pearson correlation heatmaps and Mantel tests were utilized, with data visualization performed via R.

## 3 Results

### 3.1 Soil properties and enzyme activity

Supplementary Table S1 presents the changes in the soil properties and enzyme activity. The description of the soil properties has been described in our previous study (Huang et al., 2024), and the specific results are shown in Supplementary Table S1. The enzyme activities of UR and POD varied among the three provenances, with QJ exhibiting significantly higher UR activity than SM and HF (*p* < 0.05). The order of POD activity was SM > HF > QJ, and significant differences were observed in the activity levels, with SM exhibiting a marked increase compared with that of QJ (*p* < 0.05). The activity of the Nir enzyme decreased with increasing levels of carbon sequestration of *P. massoniana*. The activity of PPO showed an increasing trend with the increase of carbon sequestration. Nir and PPO activities did not significantly differ among the three provenances.

### 3.2 Changes in microbial community structure

**Figure 1:**
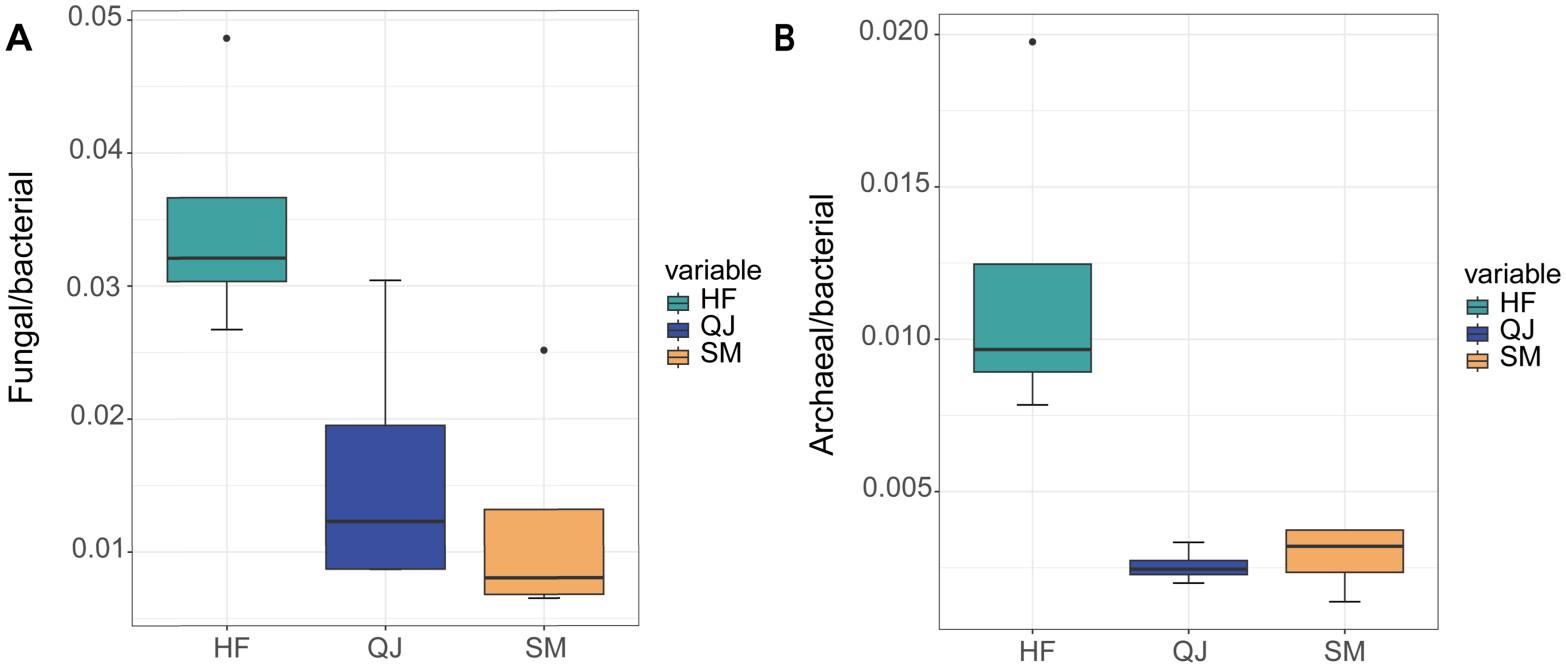
**(A)** Fungal to bacterial abundance ratios among different *P. massoniana* provenances; **(B)** Archaeal to bacterial abundance ratios among different *P. massoniana* provenances.

On average, prokaryotes accounted for 99.85% of all microbial species, with bacteria representing 97.33%, fungi accounting for 1.98%, and archaea accounting for 0.54%. The fungal to bacterial abundance ratio and the archaeobacterial to bacterial abundance ratio decreased with increasing carbon storage (Figure 1). Additionally, the values of QJ and SM were more closely aligned with those of HF.

**Figure 2:**
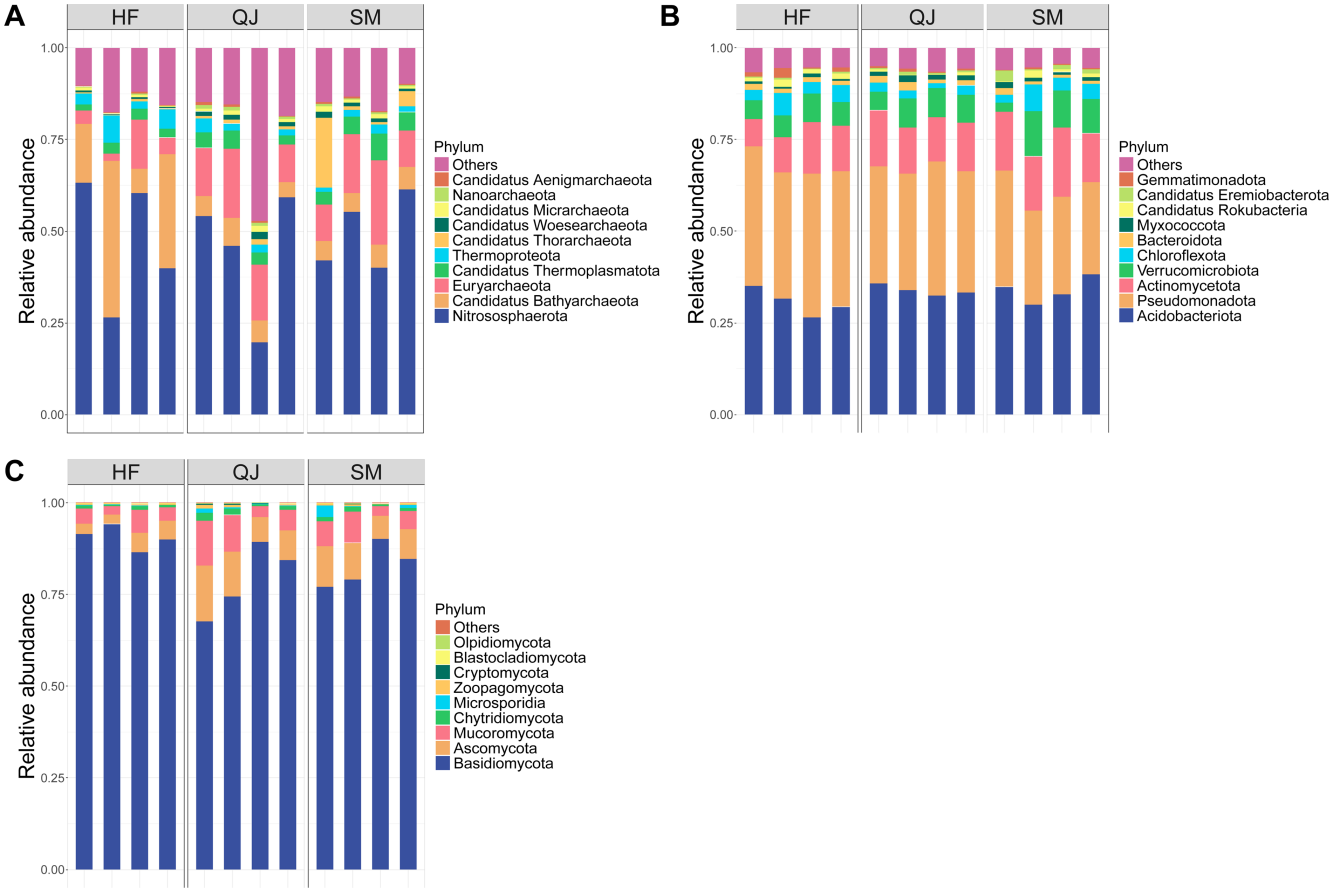
**(A)** Relative abundances of archaeal phyla associated with different *P. massoniana* provenances; **(B)** Relative abundances of bacterial phyla associated with different *P. massoniana* provenances; **(C)** Relative abundances of fungal phyla associated with different *P. massoniana* provenances.

The major phyla of soil microorganisms were *Acidobacteriota*, *Pseudomonadota*, *Actinomycetota*, and *Verrucomicrobiota* (Figure 2B). As the carbon storage level increased, the bacterial community shifted toward *Acidobacteriota*. In the low-carbon storage source, the bacterial community of HFs was predominantly composed of *Pseudomonadota*. The major fungal phyla included *Basidiomycota*, *Ascomycota*, *Mucoromycota*, and *Chytridiomycota* (Figure 2C). While the relative abundances of the dominant phyla remained unchanged, their relative abundances varied with the level of carbon storage among the provenances. Compared with those in the SM and QJ provenances, the relative abundance of *Basidiomycota* in the HF provenance was notably greater; conversely, the relative abundance of *Ascomycota* significantly differed across provenances and was notably lower in the HF provenance than in the SM and QJ provenances. *Nitrososphaerota* was the predominant archaeal community, followed by *Candidatus_Bathyarchaeota* and *Euryarchaeota* (Figure 2A). The relative abundance of *Nitrososphaerota* significantly decreased as the carbon storage level increased (*p* < 0.05), but *Nitrososphaerota* remained the primary dominant phylum.

### 3.3 Changes in genes involved in the carbon cycle

**Figure 3:**
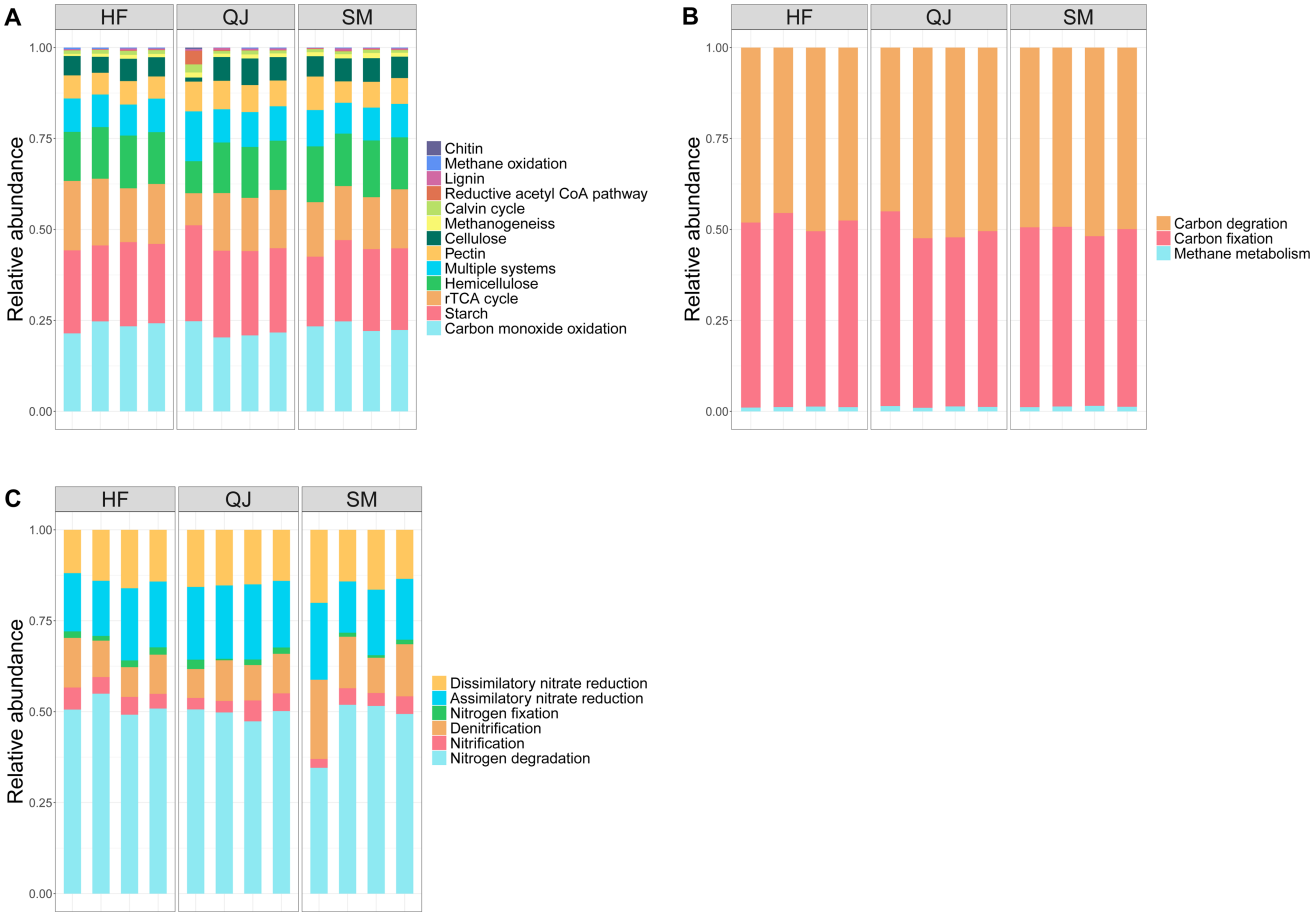
**(A)** The relative abundances of different types of carbon cycle genes (detailed classification); **(B)** The relative abundances of different types of carbon cycle genes (overall classification); **(C)** The relative abundances of different types of nitrogen cycle genes.

Genes involved in the carbon cycle can be classified into three groups: those involved in carbon degradation, those involved in carbon fixation, and those involved in methane metabolism (Figure 3). In this study, the predominant carbon cycling genes were associated with carbon degradation and fixation, whereas the methane metabolism genes presented the lowest relative abundance. In the category of carbon degradation, genes were predominantly associated with the breakdown of hemicellulose, cellulose, chitin, pectin, lignin, and starch. Among these, genes associated with the degradation of starch were the most prevalent, followed by genes associated with the breakdown of hemicellulose. Conversely, the relative abundance of genes involved in chitin and lignin degradation was relatively low. Furthermore, genes associated with carbon monoxide oxidation and diverse carbon fixation processes presented higher relative abundances than did those associated with the reductive acetyl-CoA pathway and Calvin cycle.

**Figure 4:**
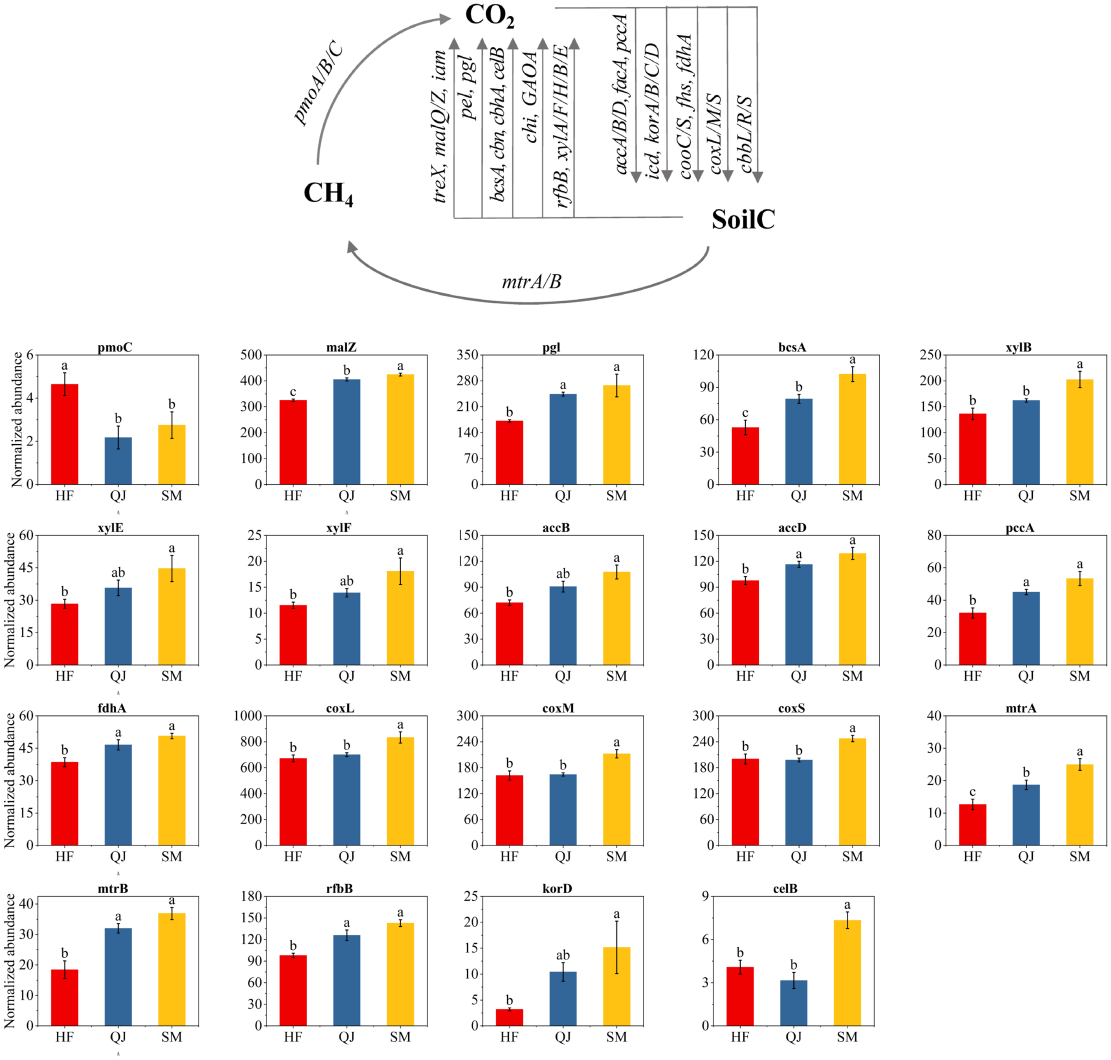
Diagram depicting the different carbon cycling processes based on metagenomic sequencing and trends in the relative abundance of gene clusters related to the soil carbon cycle.

Our ANOVA results reveal significant differences in microbial carbon cycling functional genes within the rhizosphere across the different carbon storage levels of the *P. massoniana* provenances (Figure 4). Significant changes were observed in the relative abundance of genes linked to methanogenesis (*mtrA* and *mtrB*) and methane oxidation (*pmoC*) in response to alterations in carbon storage levels; specifically, *mtrA* and *mtrB* gene abundances increased notably as carbon storage increased (*p* < 0.05), whereas *pmoC* gene abundances decreased significantly with increased carbon storage (*p* < 0.05). For the carbon fixation group, the relative abundances of the *coxL*, *coxM*, *coxS* (carbon-monoxide oxidation) and *korD* (rTCA cycle) genes increased significantly with increasing *P. massoniana* carbon storage level (*p* < 0.05). There were no significant variations in the genes associated with the reductive acetyl-CoA pathway or the Calvin cycle. The relative abundance values of genes linked to carbon degradation were similar to those in the carbon fixation group. The relative abundances of *xylB*, *xylE*, *xylF* (rTCA cycle), *celB* (cellulose), *pgl* (pectin), and *malZ* (starch) increased with increasing carbon storage level.

### 3.4 Changes in genes involved in the nitrogen cycle

The genes critical to the nitrogen cycle include those involved in nitrogen fixation, nitrogen degradation, nitrification, denitrification, dissimilatory nitrate reduction (DNRA), and assimilative nitrate reduction (ANRA) processes (Figure 3). The analysis of gene abundance revealed that among the genes sampled in this study, nitrogen degradation genes presented the greatest relative abundance values, followed by genes involved in assimilatory and dissimilatory nitrate reduction (DNRA), as well as those associated with denitrification processes. Conversely, genes related to nitrification presented the lowest abundance values.

**Figure 5:**
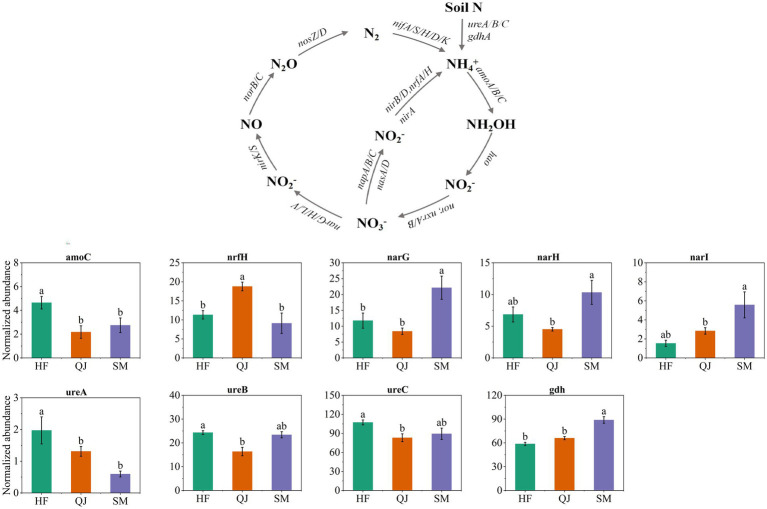
Diagram depicting the different nitrogen cycling processes based on metagenomic sequencing and trends in the relative abundance of gene clusters related to the soil nitrogen cycle.

A comparison of gene abundance related to nitrogen cycling across different samples revealed significant variations in nitrogen cycling within the rhizosphere of *P. massoniana* provenances with distinct carbon storage levels (Figure 5). Specifically, in the different *P. massoniana* provenances, an increase in carbon storage was associated with an increase in the abundance of the *gdh* gene, which is crucial for nitrogen degradation, within the rhizosphere and there were notably elevated levels in SM compared with those in both QJ and HF. Moreover, the relative abundances of the *ureA*, *ureB*, and *ureC* genes, which are also involved in nitrogen degradation, decreased notably with increasing carbon storage levels. Compared with those in the other provenances, the *ureA/B/C* genes in the HF provenance presented a notably greater relative abundance (*p* < 0.05), with significant distinctions noted for the *ureA* gene among HF, QJ, and SM. Additionally, the abundance of *amoC* genes associated with nitrification decreased with increasing carbon storage level, with significantly greater levels in HF (low carbon storage provenances) than in QJ and SM (*p* < 0.05). Furthermore, the relative abundance of the *narG*, *narH*, and *narI* genes related to denitrification increased with increasing carbon storage level, with the SM provenance (high carbon storage) exhibiting markedly elevated levels in contrast to those of the QJ and HF provenances (*p* < 0.05). Notably, among the different provenances, the prevalence of the *nrfH* gene, which play a crucial role in assimilatory nitrate reduction (ANRA), exhibited substantial variations. In this regard, the relative abundance of the *nrfH* gene in the QJ provenance was significantly greater than that in both the HF and SM provenances (*p* < 0.05). Notably, across different *P. massoniana* provenances, the levels of gene abundance related to nitrogen fixation and dissimilatory nitrate reduction (DNRA) did not show notable variations.

### 3.5 Correlation between microbial functional genes and soil factors

**Figure 6:**
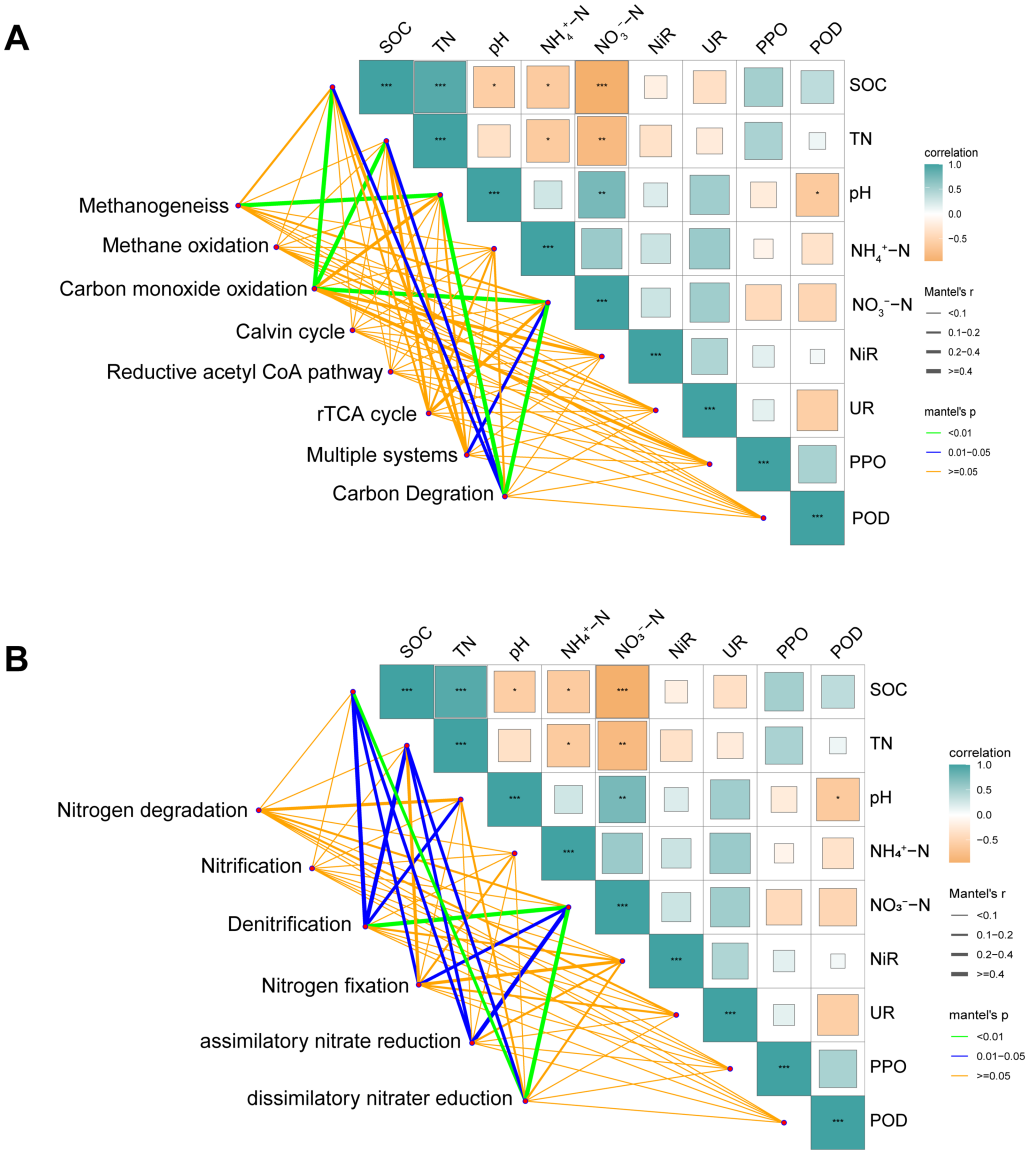
Mantel’s test for the correlations between soil properties, enzyme activity and carbon **(A)**, and nitrogen **(B)** cycling gene composition. The color gradient represents the Pearson correlation coefficient. The width of the edge reflects Mantel’s r statistic, indicating the strength of the distance correlation, whereas the edge color denotes the level of statistical significance.

Mantel tests were performed to investigate the impact of environmental variables on the functionality of microbial genes (Figure 6). The findings highlighted a notable linkage between soil environmental factors (Huang et al., 2024) and genes linked to carbon and nitrogen cycling in *P. massoniana* rhizosphere microorganisms. The SOC, TN, pH, and NO_3_^−^-N contents were the primary factors influencing carbon and nitrogen cycle genes. Specifically, the pH value predominantly influenced methane production (*r* = 0.596 *p* = 0.001); SOC (*R* = 0.59, *p* = 0.003), TN (*R* = 0.51, *p* = 0.005), and NO_3_^−^-N (*R* = 0.524, *p* = 0.003) were key factors affecting carbon monoxide oxidation; and NO_3_^−^-N (*R* = 0.275, *p* = 0.047) was the key factor affecting multiple systems. Moreover, SOC (*R* = 0.327, *p* = 0.017), TN (*R* = 0.296, *p* = 0.023), NO_3_^−^-N (*R* = 0.578, *p* = 0.001), and pH (*R* = 0.602, *p* = 0.001) primarily influenced carbon degradation. With respect to nitrogen cycle genes, the SOC (*R* = 0.431, *p* = 0.011), TN (*R* = 0.431, *p* = 0.016), NO_3_^−^-N (*R* = 0.526, *p* = 0.004), and pH (*R* = 0.255, *p* = 0.042) values were crucial for denitrification. In addition, SOC (*R* = 0.249, *p* = 0.04) and NO_3_^−^-N (*R* = 0.356, *p* = 0.025) were the main factors affecting nitrogen fixation. SOC, TN, and NO_3_^−^-N played significant roles in the assimilative and dissimilatory nitrate reduction processes. Analysis via a Pearson heatmap revealed strong correlations between most carbon cycle genes and certain nitrogen cycle genes and the corresponding soil physical and chemical properties (Supplementary Figures S1, S2). With the Notable exception of methane oxidation-related genes, carbon cycle genes (including genes related to methane production, carbon degradation, and various systems) presented positive associations with soil SOC and TN contents but negative associations with NO_3_^−^-N, NH_4_^+^-N, and pH levels. Conversely, there were inverse correlations between methane oxidation genes and soil properties. With respect to nitrogen cycle genes, the denitrification-related genes (*narI* and *narV*) were positively associated with SOC (*p* < 0.05) and negatively associated with NO_3_^−^-N and pH (*p* < 0.05). The genes related to nitrogen degradation and fixation (*amoC*, *amoA*, and *URE*) presented inverse correlations with the soil properties.

## 4 Discussion

### 4.1 Changes in the rhizosphere microbial communities of different *Pinus massoniana* provenances

Through prolonged adaptation and the forces of natural selection, the provenances of *P. massoniana*, which are characterized by varying levels of carbon storage, cultivate unique microbial communities. These communities exert a profound influence on the life-cycle ecosystems of *P. massoniana* forests (Sokol et al., 2022). Fungi play a pivotal role in the ecosystem, as their hyphae can encase the roots of trees and penetrate the soil’s mineral matrix. This allows fungi to access photosynthetic products continuously, benefiting plants by providing additional mineral nutrients through their fungal symbionts. These associations help plants combat pathogens and drought, offering a competitive advantage to both symbiotic partners (Harris, 2009). This investigation revealed that in the rhizosphere soil of *P. massoniana*, as carbon storage levels increased, there was a concurrent increase in the concentrations of SOC, TN, and additional nutrients, accompanied by an increase in fungi adept at decomposing organic matter. However, the fungal-to-bacterial ratio decreased with increasing levels of carbon storage. Bacteria excrete gums and mucilage during hyphal growth, serving as adhesives that foster mycorrhizal relationships (Harris, 2009). Consequently, the reduced fungal-to-bacterial abundance ratio found in this study suggests a more favorable environment for mycelium development. Moreover, the ratio of the abundance ratio of archaea to bacteria decreased as the carbon storage level increased in *P. massoniana* in the present study, indicating the significant role of archaea under conditions of low nutrient content (Wang et al., 2024).

In the rhizosphere of *P. massoniana*, the predominant bacterial communities belonged to the phyla *Pseudomonas* and *Acidobacteria*. In the rhizosphere, *Pseudomonas* is a group of plant growth-promoting bacteria (PGPR) that can secrete antibiotics and siderophores. It has emerged as a robust competitor in the spatial microenvironment of plants, assisting *P. massoniana* in defending against plant pathogen invasion (Chen et al., 2019). *Acidobacteria* are acidophilic bacteria and thus constitute a significant proportion of the mildly acidic rhizosphere soil of *P. massoniana* (Pankratov et al., 2012). The fungal communities in the *P. massoniana* rhizosphere samples were largely dominated by *Basidiomycota*. *Basidiomycetes* are essential for breaking down complex organic matter, with lignin being their primary degradation substrate (Riley et al., 2014). The abundance of *Basidiomycota* was greater in the rhizosphere of low-carbon-fixing sources than in that of medium- and high-carbon-fixing sources, possibly due to the elevated lignin content in the low-carbon-fixing soil sources. *Ascomycota*, a distinguished fungal phylum, significantly contributes to organic matter decomposition and the synthesis of advantageous metabolites. The increased prevalence of *Ascomycota* with increasing carbon storage levels may be linked to the heightened level of root exudates in the provenance (Yan et al., 2020). *Nitrososphaerota* emerged as the dominant archaeal community found in this investigation, which aligns with previous findings (Makk et al., 2024). This archaeal group, which is widespread across marine, freshwater, and terrestrial ecosystems, plays a pivotal role in the global cycles of carbon and nitrogen (Kim et al., 2011). The proportional abundance of *Nitrososphaerota* in the rhizosphere of *P. massoniana* decreased considerably with increasing levels of carbon storage, implying that provenance variances affect nitrification by rhizosphere microorganisms (Stieglmeier et al., 2014). Another prevailing archaeal community found in this study was *Euryarchaeota*, which exhibit a broad distribution in diverse environmental settings. *Euryarchaeota* are believed to be methanogens capable of metabolizing carbon and hydrogen under hypoxic conditions through symbiotic or fermentative lifestyles (Liu et al., 2018). Hence, it is suggested that the rhizosphere soils of *P. massoniana* plantations present the potential for more diverse carbon and nitrogen cycling. However, numerous questions remain unanswered regarding the physiology, metabolism, and ecological niche of these microbial taxa due to challenges in their isolation.

### 4.2 Changes in microbial-mediated carbon cycling patterns in the rhizosphere

In *P. massoniana* plantations, the three carbon metabolism pathways were consistent across different provenances, with an increased number of genes linked to carbon fixation and degradation and a decreased number of genes linked to methane metabolism. Our findings indicate that microbial carbon sequestration and degradation significantly contribute to the carbon cycle within the rhizosphere (Bhattacharyya et al., 2022). Furthermore, substantial variations in the gene abundance values for microbial carbon cycling within the rhizospheres of different *P. massoniana* provenances were observed, suggesting distinct functional advantages of microbial carbon cycling in the rhizospheres of different *P. massoniana* provenances. *P. massoniana* provenances with differing carbon storage capabilities impact soil microbial ecological functions by altering nutrient conditions in the rhizosphere microenvironment (Li et al., 2022). Research has suggested that carbon fixation primarily occurs through the tricarboxylic acid cycle (Li et al., 2022). Our study revealed that in the rhizosphere soil of *P. massoniana*, CO oxidation emerged as the chief pathway for carbon fixation, potentially influenced by the plant root microdomains within the rhizosphere (Jing et al., 2019). In certain polluted environments (e.g., industrial areas with CO emissions), microorganisms engaged in the CO oxidative metabolic pathway may play crucial roles in environmental remediation by utilizing CO as an energy source for growth and metabolic activities, leading to a reduction in the CO concentration in the environment and alleviating its adverse impacts on ecosystems (Han et al., 2024). Among these genes, the relative abundance values of the coxL, coxM, and coxS genes, which are associated with carbon monoxide oxidation metabolism, increased with increasing carbon storage level in the *P. massoniana* provenance, indicating that *P. massoniana* exhibiting elevated carbon storage capacities possess greater ecological restoration potential. Overall, carbon fixation strategies exhibited variability within the rhizosphere soil across various provenances of *P. massoniana* correlate with an enhancement in the carbon sink of the soil function that is parallel to the increase in carbon storage within these provenances.

Within the rhizosphere of plants, enzymes dedicated to carbon degradation breakdown organic material into small sugar molecules, thereby furnishing soil microbes with essential carbon sources and energy for their development and metabolic activities (Davidson et al., 2008). We found that the abundance of genes associated with the degradation of relatively labile carbon, such as starch (*malZ*), cellulose (*celB* and *bcsA*), and hemicellulose (*xylB*, *xylF*, *xylE*, and *rfbB*), significantly increased with increasing levels of carbon storage in the provenances. However, the abundance values of genes associated with relatively stable carbon degradation, namely, the chitin (*chi*) and lignin (*GAOA*) degradation genes, did not significantly differ among the different provenances. In this study, the results reveal that the carbon-degrading enzymes within the rhizosphere of *P. massoniana* provenance characterized by high carbon storage had a strong decomposition potential for labile carbon. Moreover, the relative abundance of *pmoC*, a gene associated with methane oxidation, showed a trend to that of other carbon cycle genes found in this study, with a significant decrease with increasing carbon storage in the *P. massoniana* provenances. Research has demonstrated that a relatively high SOC content has a certain inhibitory effect on the abundance of methane oxidation genes (Kosugi et al., 2020). In the *P. massoniana* provenances characterized by high carbon storage, an observed decline in the relative abundance values of *pmoC* genes was attributable to increased levels of nutrients and organic carbon within the rhizosphere.

### 4.3 Changes in microbial-mediated nitrogen cycling patterns in the rhizosphere

In this study, nitrogen degradation genes emerged as the most prevalent component of the nitrogen cycling process in the rhizosphere soil of *P. massoniana* plantations on the basis of the KEGG database. These findings indicated that most of the rhizosphere soil nitrogen was converted into available nitrogen for plant uptake and utilization through microbial-mediated nitrogen mineralization, underscoring the elevated fertility of the rhizosphere soil of *P. massoniana* (Hu et al., 2022a). There were significant differences in nitrogen cycling genes within the rhizosphere soil of the *P. massoniana* provenances, categorized by varying levels of carbon storage. The *ureA*, *B*, and *C* genes, which are linked to nitrogen degradation, presented significantly greater relative abundances in low-carbon storage provenances than in other provenances. Conversely, in provenances characterized by high carbon storage, the *gdhA* gene, which plays a critical role in nitrogen degradation, presented a markedly greater relative abundance than it did in the other provenances. Despite their involvement in nitrogen degradation, the *ure* and *gdh* genes catalyze different reactions. Urease, encoded by the gene *ure*, mainly catalyzes the decomposition of urea into carbon dioxide and ammonia, whereas *gdh*, encoded by the gene *GDH*, catalyzes the oxidative deamination of glutamate (Liu et al., 2024). The variability in metabolite profiles within the rhizospheres of different *P. massoniana* provenances influences the nutrient dynamics of these zones, subsequently altering the distribution of functional gene abundances (Li et al., 2022).

With increasing carbon storage, we observed a notable decrease in the relative abundance of the *amoC* gene, which is crucial for nitrification processes, whereas the abundance of genes linked to denitrification, namely, *narG*, *narH*, and *narI*, noticeably increased. We posit that, owing to the low nutrient level within the rhizosphere combined with nitrogen scarcity in the soil, low-carbon storage provenances might acquire as much nitrogen as possible, leading to changes in the gene functions of microorganisms and enabling these low-carbon storage provenances to maintain normal growth (Wang et al., 2024). However, in provenances with lower carbon storage, an elevated abundance of genes associated with nitrification in the rhizosphere may increase NO_3_^−^-N accumulation in the soil, which could increase the risk of nitrogen depletion by leaching and runoff (Qi et al., 2020). In *P. massoniana* provenances with high carbon storage, an increased potential for denitrification mitigated nitrite nitrogen buildup in the soil, abated the risk posed by excessive nitrate levels, and contributed beneficially to the vitality of the soil biota and overall ecosystem health (Dong et al., 2020). In provenances with a medium carbon storage level, a notable increase in the relative abundance of the *nrfH* gene, which is crucial for the assimilatory reduction of nitrate (ANRA) within the rhizosphere nitrogen cycle, was observed compared with that in provenances with other carbon storage levels. ANRA can promote the accumulation of NH_4_^+^, preserve more reactive nitrogen for ecosystems, and promote denitrification (Hidalgo-Garcia et al., 2019). Therefore, we speculate that provenances with intermediate levels of carbon storage has a strong ability to retain inorganic nitrogen within the rhizosphere, facilitating plant growth and development.

### 4.4 Main environmental factors influencing the processes of carbon and nitrogen cycling

Our previous biological-eco experimental study revealed significant differences in the rhizosphere microbial carbon metabolism capacity of *P. massoniana* provenances characterized by varying degrees of carbon storage (Huang et al., 2024). In this research, metagenomic approaches were employed to explore the genes associated with differential carbon and nitrogen cycling within the rhizosphere of *P. massoniana* in detail. Mantel and Pearson correlation analyses revealed that genes linked to the carbon and nitrogen cycles presented positive or negative correlations with soil properties. The functional genes linked to carbon fixation and carbon degradation were positively correlated with the contents of SOC and TN. Under conditions of abundant organic carbon and plentiful nutrients, soil microorganisms accelerate the utilization of carbon sources because of their rapid growth rate, resulting in elevated gene abundance associated with carbon fixation and degradation (Ma et al., 2022). Methane metabolism was inversely correlated with the SOC and TN contents, which is in agreement with the findings of other studies (Kosugi et al., 2020). Methanogen-induced methane production rates were negatively correlated with the SOC content. Additionally, Zhao Jiao et al. demonstrated a significant negative relationship between the abundance of functional genes for methane production (*mxa* and *emGDH*) and methane oxidation (*pmoA* and *mmoX*) in soil undergoing vegetation restoration with SOC and TN contents. Certain methane-metabolizing microbial populations living in nutrient-poor environments with low carbon and nitrogen levels exhibit strong oxidative potential and a high abundance of methane oxidation genes (Zheng et al., 2010). Consequently, as the levels of soil nutrients and organic carbon fractions in the rhizosphere decreased alongside the reduction in carbon storage in *P. massoniana*, the abundance of functional genes linked to methane metabolism increased.

Our findings confirm that the SOC and NO_3_^−^-N contents are key soil factors influencing nitrogen cycling genes within the rhizosphere of *P. massoniana*. Genes linked to nitrogen degradation and denitrification presented significant positive correlations with the SOC content and negative correlations with the NO_3_^−^-N level. Conversely, the abundance of nitrification-associated genes inversely correlates with SOC levels but positively aligns with NO_3_^−^-N concentrations. This suggests that soil organic carbon acts as the main carbon source that fuels microbial processes in the soil (Chen et al., 2024). Microbial taxa pertinent to this process degrade organic matter during nitrogen degradation to acquire energy and nutrients, including carbon sources. Furthermore, denitrification, for the most part, is facilitated by heterotrophic bacteria that depend on organic carbon in the soil as an energy and carbon source. Thus, genes related to nitrogen degradation and denitrification are positively correlated with the soil organic carbon content (Wang et al., 2023). Nevertheless, a competitive relationship exists between these heterotrophic microorganisms and autotrophic nitrifying bacteria. Heterotrophic microorganisms utilize organic carbon for growth and reproduction, whereas nitrifying bacteria primarily derive energy from oxidizing inorganic nitrogen such as ammonia. Hence, a notable inverse correlation between nitrification-related genes and the soil NO_3_^−^-N content was identified in this study (Wang et al., 2022). In general, with elevated soil organic carbon content, the competitive edge of heterotrophic microorganisms is enhanced, leading to the presence of genes associated with denitrification. Simultaneously, they compete with nitrifying bacteria for resources such as dissolved oxygen and substrates (such as ammonia), ultimately restraining the growth and activity of nitrifying bacteria and reducing the expression and abundance of nitrification-related genes.

Clearly, shifts in the provenances of *P. massoniana* lead to changes in both the litter composition and the root exudates and profiles, consequently modifying the nutrient composition of the soil. These alterations significantly affect the configuration of the soil microbial community structure as well as the ecosystem services they underpin (Narula et al., 2009). Soil microorganisms are crucial for promoting the development of *P. massoniana* and preserving the balance of the plantation ecosystem by adapting effectively (Huang et al., 2023). Understanding the shifts in microbial community structure and functionality is crucial for comprehending their potential responses to the varying carbon storage capacities across *P. massoniana* provenances. SM, a *P. massoniana* provenance characterized by high carbon storage, presented superior rhizosphere microbial carbon and nitrogen cycling capabilities along with greater potential for environmental remediation.

## 5 Conclusion

In this research, notable discrepancies were observed in the abundance of functional genes linked to carbon and nitrogen cycling within the rhizosphere soil of *P. massoniana* provenances, which presented varying levels of carbon storage. The carbon fixation of the rhizosphere soil occurred primarily through the carbon monoxide oxidation metabolic pathway, indicating potential environmental remediation capabilities in *P. massoniana* plantations. The abundance of genes linked to carbon degradation, carbon fixation, methane production, and denitrification in the rhizosphere of *P. massoniana* with high carbon storage levels significantly surpassed those of *P. massoniana* with moderate and low carbon storage levels, thereby notably enhancing the conversion and availability of soil carbon and nitrogen within *P. massoniana* plantations. Provenance SM, recognized for its high carbon storage ability, exhibited superior rhizosphere microbial carbon cycling and denitrification functions, alongside heightened potential for ecological restoration. Therefore, SM provenance can be promoted and planted as an excellent provenance with high ecological value in the Anhui region of China.

## Data Availability

The datasets presented in this study can be found in online repositories. The names of the repository/repositories and accession number(s) can be found in the article/Supplementary material.
