# Supplementary material for: Divergent profiles of rhizosphere soil carbon and nitrogen cycling in Pinus massoniana provenances with different types of carbon storage
Source: Front Microbiol. 2025 Mar 17;16:1537173. doi: 10.3389/fmicb.2025.1537173 (PMC11955601; doi:10.3389/fmicb.2025.1537173)
Supplement: Supplementary file 1 [file Data_Sheet_1.docx]

**Supplementary Materials**

**Table of Contents:**

**Figure S1. Heat maps showing the Pearson correlations between the soil properties, enzyme activity and carbon cycle genes.**

**Figure S2. Heat maps showing the Pearson correlations between the soil properties, enzyme activity and nitrogen cycle genes.**

**Table S1. Soil properties and enzyme activities in rhizosphere of different *Pinus massoniana* provenances**

**Table S2.** **Information of microbial functional genes involved in the C and N cycling processes identified in this study.**

**Table S3.** **Results of Mantel tests between carbon and nitrogen cycling genes composition with environmental factors across all treatment, values at p<0.05 are shown in bold.**

**Text S1. Measurements of soil properties**


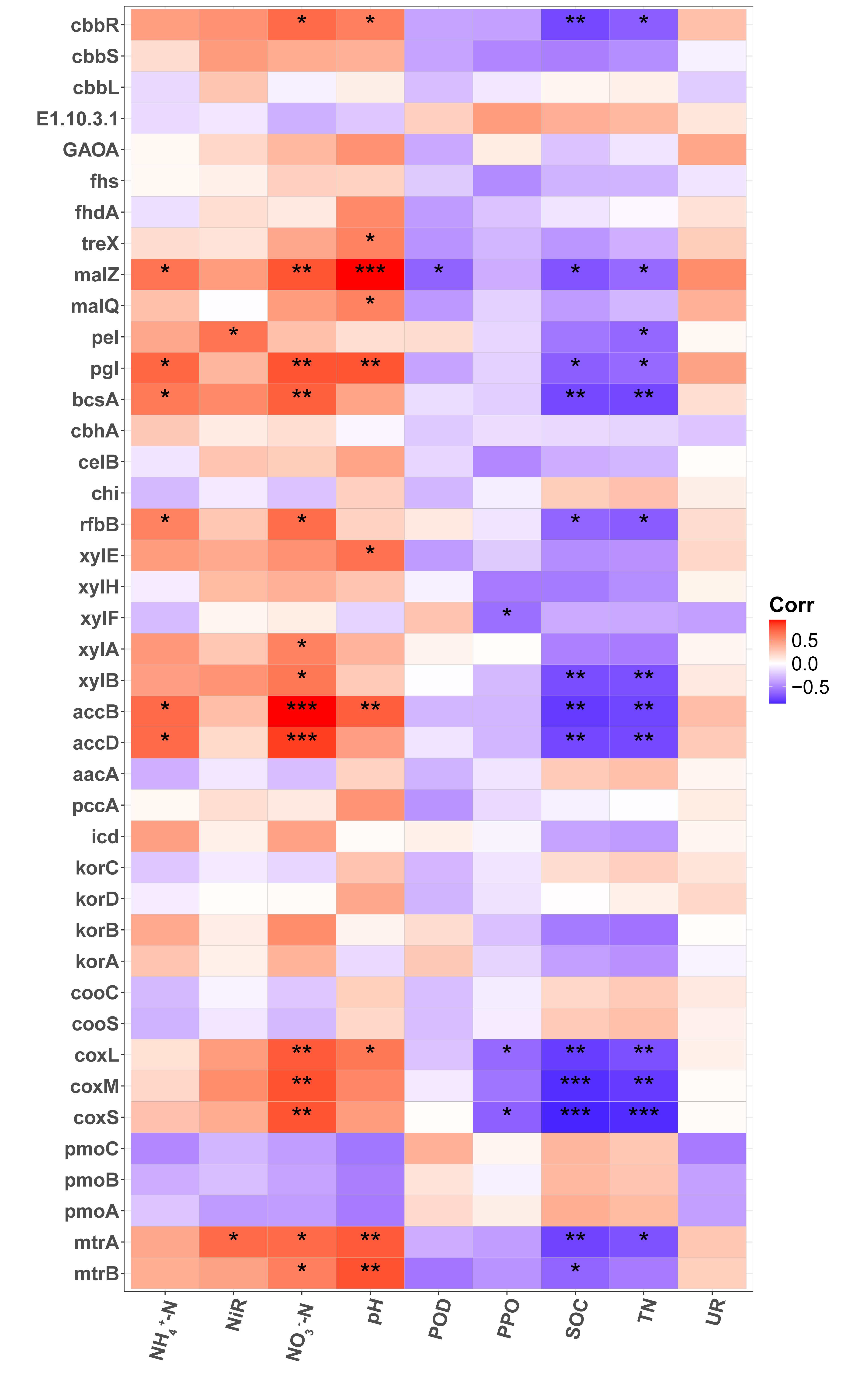


**Figure S1. Heat maps showing the Pearson correlations between the soil properties, enzyme activity and carbon cycle genes.**


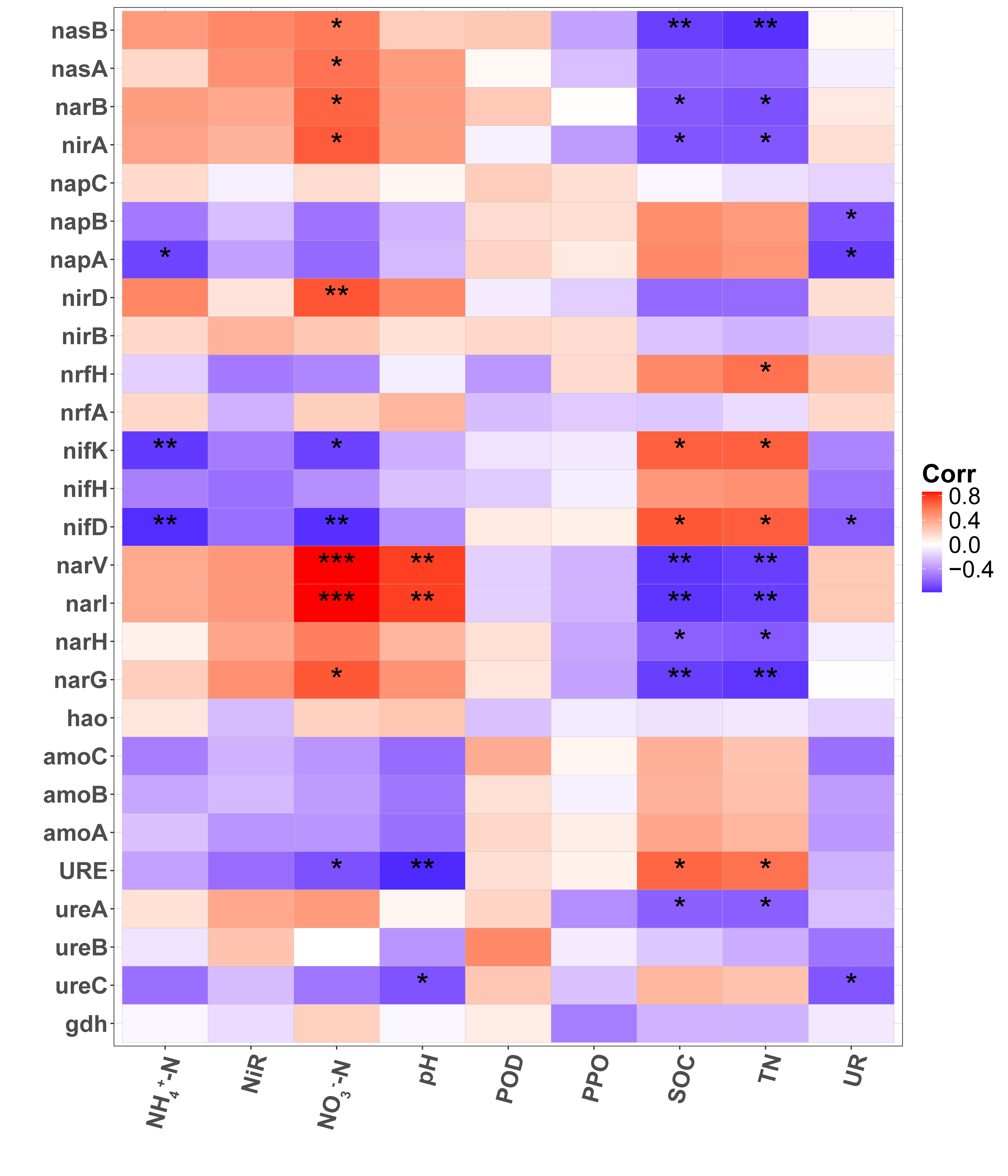


**Figure S2. Heat maps showing the Pearson correlations between the soil properties, enzyme activity and nitrogen cycle genes.**

**Table S1. Soil properties and enzyme activities in rhizosphere of different Pinus massoniana provenances**

|  | SM | QJ | HF |
| --- | --- | --- | --- |
| SOC(g/kg) | 27.59±0.74a | 25.11±1.42a | 12.29±0.29b |
| TN(g/kg) | 2.61±0.06a | 2.59±0.13a | 1.25±0.07b |
| pH | 4.61±0.02b | 4.85±0.01a | 4.89±0.04a |
| NH4+-N(μg/g) | 6.11±0.75b | 9.23±1.56ab | 10.66±1.18a |
| NO3--N(μg/g) | 0.99±0.43c | 5.42±1.34b | 11.68±1.41a |
| NiR(μmol/d/g) | 20.62±1.15 | 20.31±1.48 | 29.65±4.68 |
| UR(μmol/d/g) | 131.78±28.67b | 238.93±33.09a | 193.16±21.62ab |
| PPO(nmol/h/g) | 270.26±25.71 | 262.14±36.82 | 204.04±11.11 |
| POD(nmol/h/g) | 2820.06±135.45a | 1482.32±327.96b | 2134.98±114.17ab |

Note: SM, SanMing provenance; QJ, QingJiang provenance; HF, HeFeng provenance; Different letters indicate significant differences among provenances using one-way ANOVA (LSD, P < 0.05).

**Text S1. Measurements of soil properties**

Soil pH was determined using the glass electrode method at a water to soil ratio of 1:2.5 (Wang et al., 2024). Soil organic carbon was determined by external heating with potassium dichromate (Benbi, 2018). Soil total nitrogen was determined by Kjeldahl method. Soil nitrate nitrogen and ammonia nitrogen were measured by flow analyzer (Bunch and Bernot, 2012).

Benbi, D. K. (2018). Evaluation of a rapid microwave digestion method for determination of total organic carbon in soil. Communications in Soil Science and Plant Analysis. 49: 2103-2112. <http://10.1080/00103624.2018.1495732>.

Bunch, N. D., Bernot M. J. (2012). Nitrate and ammonium uptake by natural stream sediment microbial communities in response to nutrient enrichment. Research in Microbiology. 163: 137-141. <http://10.1016/j.resmic.2011.11.004>.

Wang, A., Zhang Y., Wang G., Zhang Z. (2024). Soil physicochemical properties and microorganisms jointly regulate the variations of soil carbon and nitrogen cycles along vegetation restoration on the Loess Plateau, China. Plant and Soil. 494: 413-436. <http://10.1007/s11104-023-06290-2>.
